# Optimizing corn productivity: Hybrid and intra-row spacing effects on growth, yield, and nutritional quality

**DOI:** 10.1038/s41598-025-19439-z

**Published:** 2025-09-18

**Authors:** Asmaa Hamoda, Mokhtar Dabbour

**Affiliations:** 1 Department of Agronomy, Faculty of Agriculture, Benha University, P.O. Box 13736, Moshtohor, Qaluobia Egypt; 2 Department of Agricultural and Biosystems Engineering, Faculty of Agriculture, Benha University, P.O. Box 13736, Moshtohor, Qaluobia Egypt

**Keywords:** Corn hybrids, Chlorophyll content, Leaf area index, Grain yield, Carbohydrate content, Protein content, Plant sciences, Plant development, Plant physiology

## Abstract

Ongoing genetic enhancements in corn hybrids for high plant density tolerance compel agronomists to periodically reassess optimal intra-row spacing. Accordingly, this investigation assessed the growth, yield, and chemical properties of three commercial hybrids (namely Pioneer 3444, Hytech 2031, and Giza 168) sown at varied intra-row spacings (15–35 cm). Results revealed that Pioneer 3444 (V1) had observably higher chlorophyll content and leaf area index than Hytech 2031 (V2) and Giza 168 (V3), whereas significant decreases in duration to 50% tasseling and silking were documented (p < 0.05). Reference to V1 and V3, the V2 hybrid exhibited higher nitrogen and protein content (2.01 and 11.53%, respectively). Notably, the optimum intra-row spacing was noticed at 25 cm, showing significant improvements (p < 0.05) in shelling percentage (82.72%), weight of 100-grain (41.39 g), grain yield (7549.78 kg/ha), and carbohydrate (83.40%), nitrogen (2.15%), and protein content (12.24%) relative to other spacings. Moreover, the V3 hybrid grown at 35 cm spacing extended the time to 50% tasseling (70.67 days) and increased number of grains per row (46.67). Among all hybrid-spacing interactions, the V1 sown at 25 cm produced the highest (p < 0.05) ear diameter (5.73 cm), 100-grain weight (44.50 g), and grain yield (8206.87 kg/ha). Most importantly, the synergistic effect of V2 × 25 cm spacing resulted in the greatest nitrogen (2.27%) and protein (12.93%) contents, closely followed by those of V1 under the same 25 cm spacing (2.25 and 12.80%, respectively), whereas V1 × 25 cm spacing significantly maximized carbohydrate content (85.00%). Correlation analysis illustrated that the reduction in duration to 50% tasseling and silking accounted for 65–90% of the increase in ear diameter, 100-grain weight, and grain yield. These findings demonstrate that farmers should cultivate the V1 hybrid at a 25 cm spacing to achieve an optimal balance between grain yield and quality, thereby maximizing corn productivity and potential profitability.

## Introduction

The continuously growing awareness of the functional and nutritional benefits of corn (*Zea mays* L.) by the global populace has led to a corresponding increase in its use in human diets. Corn, currently the world’s highest-producing cereal crop, is expected to dominate the global cultivation and trade in the next few decades. It is largely cultivated across five continents (Asia, Europe, Africa, and North and South America) with a total area of 208.2 million ha and production of 1241.6 million tons in 2023^1^. This crop is a key source of food and livelihood for millions in many countries worldwide. Aside from having high starch content (~ 72%), corn is rich in protein^2^, essential minerals (magnesium, phosphorus, and potassium), dietary fiber, and fat, providing an energy of 365 kcal/100 g^3^. Moreover, corn grains are mainly used in the preparation of food items (e.g., starch, glucose, sweeteners, edible oil, beverages), animal and poultry feeds, and industrial materials (e.g., glue, biofuels)^4–6^. Globally, the average productivity of corn is relatively low, averaging approximately 5.96 t/ha. This low yield is often due to inappropriate intra-row plant spacing and poor agricultural management practices, which can cause significant grain yield losses for corn growers^7^, with global estimates reaching about 20%^8^. Intra-row spacing is one of the key factors that affect corn yield and its components such as number of ears, grain weight, number of grains per ear, and ear diameter and length. Unlike other members of the grass family, corn yield exhibits greater sensitivity to changes in plant population (directly associated with variations in intra-row spacing), mostly ascribed to its limited tillering ability, short flowering period, and monoecious flower structure^9,10^. Intra-row spacing critically influences resource utilization (e.g., light, water, and nutrients), which directly affects crop growth and development, especially plant height, leaf area index (LAI), root density and length, and yield components^11^. In that sense, establishing optimal spacing is essential to maximize corn growth and yield. Overly narrow spacing depletes soil nutrients and moisture before crop maturity and intensifies competition among plants for available photosynthetic photon flux density^12^, whereas excessively wide spacing wastes available rhizosphere resource and promotes weed growth, both of which reduce productivity. Plensicar and Kustori^13^ found that a dense plant population significantly augmented the susceptibility of crop to lodging, pest, and disease, concurrently reducing ear size and grain yield. Temesgen^14^ indicated that corn sown at narrower intra-row spacing (20 cm) achieved the highest LAI (3.975) and plant height (257.8 cm), while wider spacing (35 cm) maximized number of ears per plant and ear length and diameter. Additionally, higher grain and dry biomass yields were recorded at intermediate and closer plant spacing, respectively^15^. Importantly, the optimal intra-row spacing for maximizing grain yield varies across hybrids due to the interaction between plant spacing and hybrid-specific traits. Consequently, tailoring plant spacing to specific corn hybrids is crucial for achieving maximum yield potential.

Corn genotypes exhibit diverse physiological and morphological traits, necessitating optimal intra-row spacing for maximum yield. For corn hybrids, yield responds curvilinearly to intra-row spacing, peaking at the optimum spacing^16^. Modern hybrids tolerate stress and interplant competition more effectively, enabling producers to reduce intra-row spacing and maximize corn grain yields^17^. In this regard, the selection of suitable hybrids is crucial to substantially increase corn productivity^18,19^. Earlier reports demonstrated that hybrids vary markedly in their response to management factors e.g., plant spacing^20^. Koirala et al.^21^ illustrated that corn hybrids produced higher grain yields than local cultivars, primarily linked to their effectiveness in transferring assimilates to the ear sink^22^. Alias et al.^23^ noticed a significant variation between corn hybrids in number of ears per plant, plant height, LAI, number of grains per row, grain yield per plant, and grain weight per ear. Furthermore, Dwipa et al.^24^ realized that Pioneer P35 produced more leaves per plant, longer ears, and higher grain yield than Pertiwi 3, while exhibiting lower plant height, later silking, smaller ear diameter, and fewer rows per ear.

Despite the critical importance of intra-row spacing for corn hybrids, the majority of breeders and farmers remain poorly informed about essential crop management strategies, particularly optimal plant spacing and appropriate hybrid selection. Furthermore, no prior studies have comprehensively integrated growth, yield, and nutritional quality for corn hybrids (specifically Pioneer 3444, Hytech 2031, and Giza 168) under varying spacings, nor explored their physiological linkages via Pearson’s correlation and principal component analysis (PCA). Therefore, this investigation aimed to evaluate these three commercially available hybrids to examine the influence of intra-row spacing on their growth, yield, and quality attributes. Additionally, Pearson’s correlation and PCA were performed to establish the interrelationship between studied traits of corn hybrids. The findings of this study could contribute to the improvement of farmer livelihoods, but large-scale field validation is required to substantiate their economic impacts.

## Materials and methods

### Description of experimental site

**Fig. 1:**
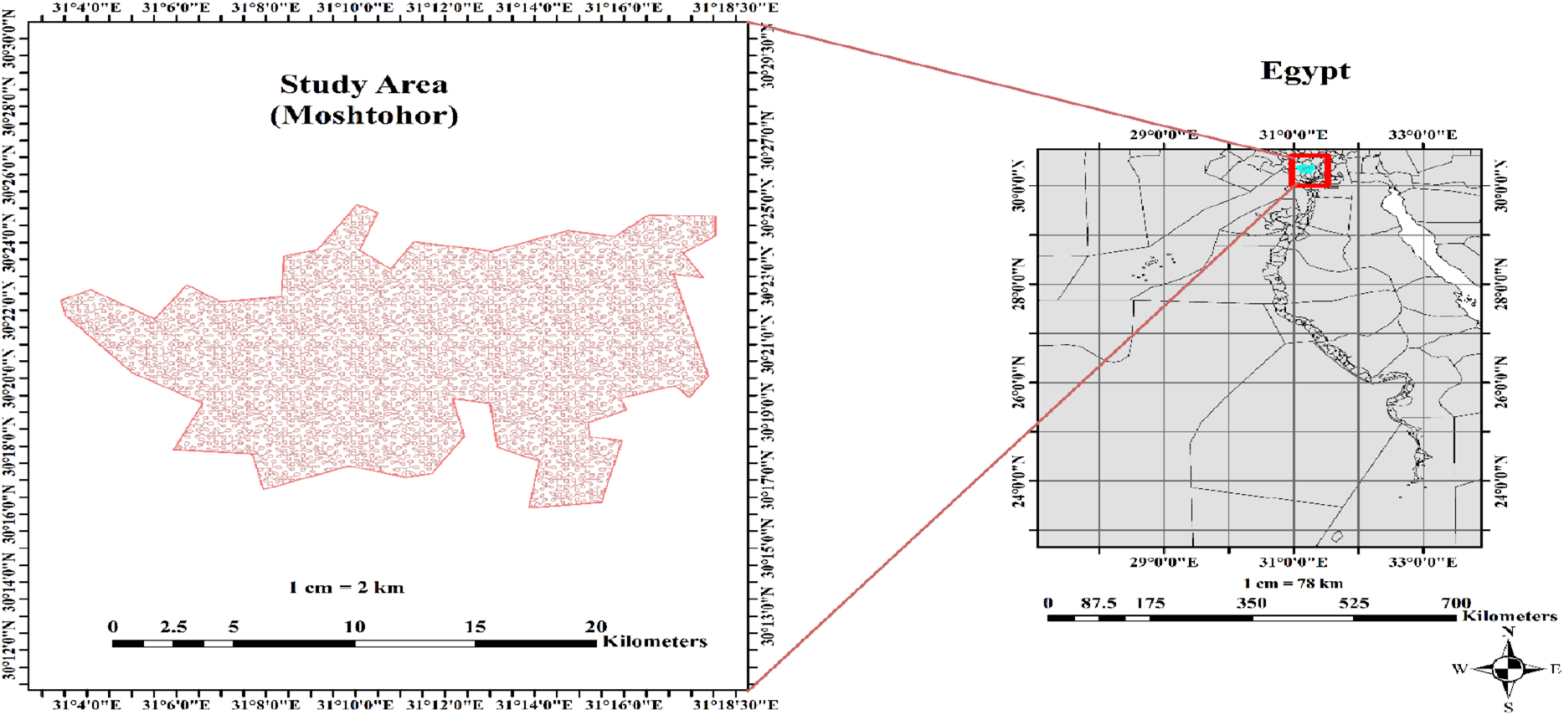
Geographical location of the experimental site (generated by ArcGIS 10.8 software).

**Fig. 2:**
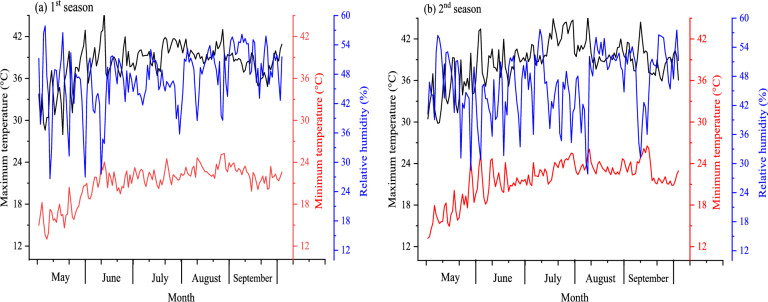
Meteorological data of the experimental site during two seasons (source: https://power.larc.nasa.gov/data-access-viewer/*)*.

Field trials were conducted at the Research and Experimental Station, Faculty of Agriculture, Benha University, Egypt (31.10° E longitude and 30.45°N latitude) over the two consecutive growing seasons of 2022 and 2023. The geographical location of the experimental site is depicted in (Fig. 1). Before the experiment, soil samples were collected using a spiral auger at two depths of 0–30 and 30–60 cm from the experimental plots, and three subsamples were taken from each plot and subsequently composited. The composited samples were dried, milled, and thereafter sieved through a 2-mm mesh for analysis. Soil pH was measured using a pH meter. Particle size distribution of soil samples was examined according to Klute^25^. Electrical conductivity (EC) was determined as described by Page et al.^26^. The cation exchange capacity (CEC) and organic matter (OM) content were analyzed following the standard analytical procedures outlined by Jackson^27^. Table 1 presents the physico-chemical attributes of experimental site. The experimental site is classified as loamy clay soil with high OM content (15.80–21.00 g/kg). The pH and CEC of soil samples ranged from 7.10–7.90 and 39.80–41.06 mol_c_ kg^-1^, respectively, in both seasons. Particle size analysis exhibited the predominance of clay fractions (52.00–55.60%), with lower amounts of sand (32.50–33.00%) and silt (11.40–14.21%). The soil possessed low EC (≤ 0.75 dSm^-1^ in both seasons), suggesting minimal salinity issues. The cation exchange was dominated by calcium (Ca^+^⁺: 2.72–3.15 mmole_c_/L), while potassium concentration was low (K⁺: 0.84–1.41 mmole_c_/L). Bicarbonate (HCO₃⁻) and chloride (Cl⁻) concentrations were 1.96–2.81 and 1.48–2.45 mmole_c_/L, respectively. Additionally, the meteorological data (i.e., relative humidity and minimum/ maximum temperature) of the study site during both growing seasons are displayed in (Fig. 2). The mean minimum air temperature from May to September was 21.06 °C in 2022 and 21.67 °C in 2023, whereas the mean maximum temperature reached 38.13 °C and 38.67 °C during the same period. Additionally, relative humidity averaged 47.04% in 2022 and 46.42% in 2023 throughout these months.

**Table 1:** Physico-chemical characteristics of experimental site during 2022/2023 seasons. OM, EC, and CEC represent the electrical conductivity, organic matter, and cation exchange capacity respectively.

Properties	2022		2023	
	0–30 (cm)	30–60 (cm)	0–30 (cm)	30–60 (cm)
EC (dSm^−1^)	0.56	0.74	0.59	0.75
pH (1:2.5 w/v)	7.10	7.24	7.36	7.90
OM (g/kg)	15.80	20.60	16.21	21.00
CEC (mol_c_.kg^−1^)	40.00	40.32	39.80	41.06
Soluble cations (mmole_c_/L)				
Ca^++^	2.90	2.72	3.00	3.15
K^+^	0.84	1.41	0.94	1.15
Soluble anions (mmole_c_/L)				
Cl^−^	1.89	2.45	1.48	2.28
CO_3_^−^	0.00	0.00	0.00	0.00
HCO_3−_	2.81	1.97	2.57	1.96
Particle size distribution (%)				
Sand	32.50	33.00	32.85	32.80
Silt	13.26	11.40	14.21	14.20
Clay	54.24	55.60	52.00	53.00
Textural class*	CLAY	clay	Clay	Clay

### Experimental design and treatments

**Fig. 3:**
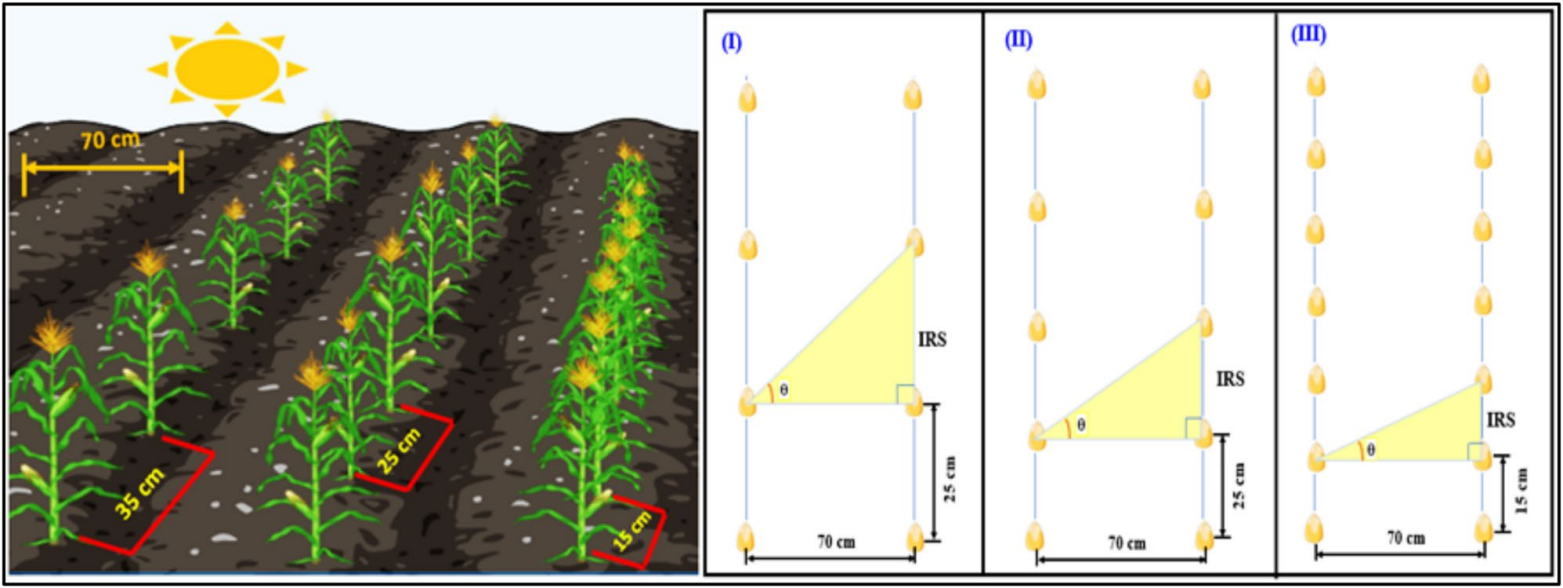
Schematic representation of intra-row spacing and angle per plant in corn cultivation (constructed by Bio-Render software).

The experiment was conducted in a Randomized Complete Block Design (RCBD) with a 3 × 3 factorial arrangement and three replications during both growing seasons, totaling 27 experimental plots. Three corn hybrids, Pioneer 3444 (V1), Hytech 2031 (V2), and Giza 168 (V3) (Table 2), were assessed under three intra-row spacing levels (15, 25, and 35 cm) (Fig. 3). The experimental plot area was 10.50 m^2^ (3.5 m width × 3.0 m length), containing 5 rows with an inter-row spacing of 0.7 m. The two outer rows (1^st^ and 5^th^) in each plot were used as borders, while the remaining rows were designated as net plot for sampling. Blocks were spaced 1 m apart, whilst an open space (1 m) was maintained between plots within blocks to facilitate field management.

**Table 2:** The designation, origin, and genetic constitution of corn hybrids.

Genotype	Designation	Origin	Genetic constitution
1	V1	Corteva Pioneer, USA	Single cross
2	V2	HYTECH, Egypt	Single cross
3	V3	Agricultural Research Center ARC, Egypt	Single cross

### Experimental field management

The trial field was ploughed, disked, leveled (to ensure a smooth seedbed), ridged, and thereafter divided into plots. Calcium superphosphate (15.5% P_2_O_5_) was uniformly broadcasted across all plots at the recommended rate of 357 kg/ha before sowing. Seeds were sown (two seeds per hole) on May 23^rd^ and 26^th^ in two growing seasons and covered with soil. Thinning was performed manually to maintain a single healthy plant per hill at the 3–4 leaf stage before the first irrigation. Nitrogen fertilizer in the form of granular urea (46.0% N) at a rate of 286 kg/ha, placed at a depth of 3–5 cm from the root zone of plants via side dressing method, was applied in two equal doses before the first and second irrigation. In this research, potassium (K) fertilization was omitted based on soil test results showing extractable K⁺ concentrations of 0.84–1.41 mmolₑ L^−1^ (equivalent to 37–55 mg kg^−1^). These values fall within established sufficiency ranges for corn (typically 30–60 mg kg^−1^ exchangeable K) and well exceed critical deficiency thresholds. Hoeing was uniformly conducted before the first and second irrigation for all plots to control weeds. The irrigation (utilizing furrow irrigation method) was continued throughout the growing seasons according to crop necessity. The plants were harvested manually at physiological maturity in September of both seasons.

### Data collection and analysis

#### Growth parameters

##### Plant height (cm)

The mean height of five randomly selected plants per plot was measured using a measuring tape, from the soil surface to the base of the tassels.

##### Number of leaves per plant

The leaves were counted from the selected plants and afterward averaged to quantify the number of leaves per plant.

##### Leaf area index (LAI)

The leaf area was initially measured from five randomly chosen plants cultivated in the net plot using plant canopy analyzer (CI-203 Handheld Laser Leaf Area Meter, USA). Subsequently, LAI was computed as follows:

$$LAI=\frac{Total leaf area \left({cm}^{2}\right)}{Ground covered area \left({cm}^{2}\right)}$$

##### Chlorophyll content

Chlorophyll content of ear-positioned leaves was analyzed by a Soil Plant Analysis Development (SPAD) chlorophyll meter (502-SPAD, Minolta, Japan) on five randomly taken plants.

##### Days to 50% tasseling

The duration (in days) from sowing until 50% of plants in each plot-initiated pollen shedding from the main tassel branches was recorded visually and used for analysis.

##### Days to 50% silking

The period (in days) from sowing until 50% of plants in each plot extruded silk was determined via visual observation.

#### Yield parameters

Five corn plants from the net area of each plot, at harvest, were randomly selected, and the yield attributes were assessed as follows:

##### Ear length

Following husk removal, ear length was measured from the base to the tip of randomly selected ears using a vernier caliper; and the mean value was then recorded.

##### Ear diameter

The randomly selected ears were dehusked; and the diameter was estimated at the center of each ear utilizing a vernier caliper. The values were averaged and then documented.

##### Number of grains per row

It was determined by averaging the grain number in each row of randomly sampled ears.

##### Weight of 100-grain (g)

One hundred grain was first counted using a GA-234-A automatic seed counter (Green Agritech Equip. Ltd., India) from the selected ears, and then weighed with a digital balance (YHC weighing excellence, Wonderscales, China).

##### Grain yield (kg/ha)

Ears from the central three rows of each plot were harvested and afterward dehusked. After sun drying, the ears were shelled using a corn sheller (Corn Thresher Maize Sheller Machine, Xiamen Great Bond Technology Co. Ltd., Fujian, China) and thereafter winnowed in the air to obtain clean grains. The grains were weighted (at 14% moisture content); and the yield of each plot was computed (kg/plot) and subsequently converted to kg/ha.

##### Shelling percentage (%)

This was calculated as the ratio of the weight of shelled grains from selected ears to the total weight of those ears, multiplied by 100.

### Chemical analysis

#### Nitrogen and protein content

Kjeldahl method was used to determine nitrogen and protein content of corn grains. The grains were oven-dried (70 °C, 48 h) and pulverized to pass through a 0.50 mm sieve. A 0.2 g of the sample powder was digested with sulfuric and perchloric acids at 380 °C, and afterward diluted to 50 mL using distilled water. A 10 mL aliquot of the diluted suspension was distilled with NaOH (20 mL). The ammonia trapped during distillation was collected in a flask having 25 mL of boric acid and two drops of indicator. The solution was then titrated using HCl (0.1 N). The nitrogen content was determined as previously detailed^28^. Protein content was then calculated by multiplying the nitrogen content by a conversion factor of 5.70.

#### Total carbohydrates

The protocol of Nielsen^29^ was followed to quantify carbohydrate content of corn grains. A 0.1 g of the sample powder was weighed, mixed with 2.5 N HCl (5 mL), and heated in water bath for 3 h. The resultant was neutralized using sodium carbonate and then made up to 100 mL with distilled water. This mixture was mixed with phenol solution (1 mL, 5%) and sulphuric acid solution (5 mL, 96%) and then allowed to react at 30 °C for 20 min. Absorbance was analyzed spectrophotometrically at 490 nm, and the total carbohydrate content was thereafter quantified. Glucose was utilized as the standard.

### Data analysis

A two-way ANOVA, consistent with the factorial experimental design arranged in an RCBD, was performed using COSTAT-V6.311 software (Cohort Software, Berkeley, CA, USA) and significant differences among means were established using Tukey’s comparison test (p < 0.05) according to Snedecor and Cochran^30^. Combined analysis over both seasons was conducted after assessing the homogeneity of error variances, as outlined by Gomez and Gomez^31^. Results are presented as means ± standard deviation. Pearson’s correlation analysis and PCA were performed using OriginPro-2023b software (OriginLab Corporation, Northampton, MA, USA) to examine interrelationships among the measured parameters of the corn hybrids under varied intra-row spacings. COSTAT software was selected for its comprehensive suite of design and statistical tools specific to agricultural field experiments, while OriginPro was used for its powerful graphical capabilities and efficiency in generating publication-ready figures.

## Results and discussion

### Plant height

**Fig. 4:**
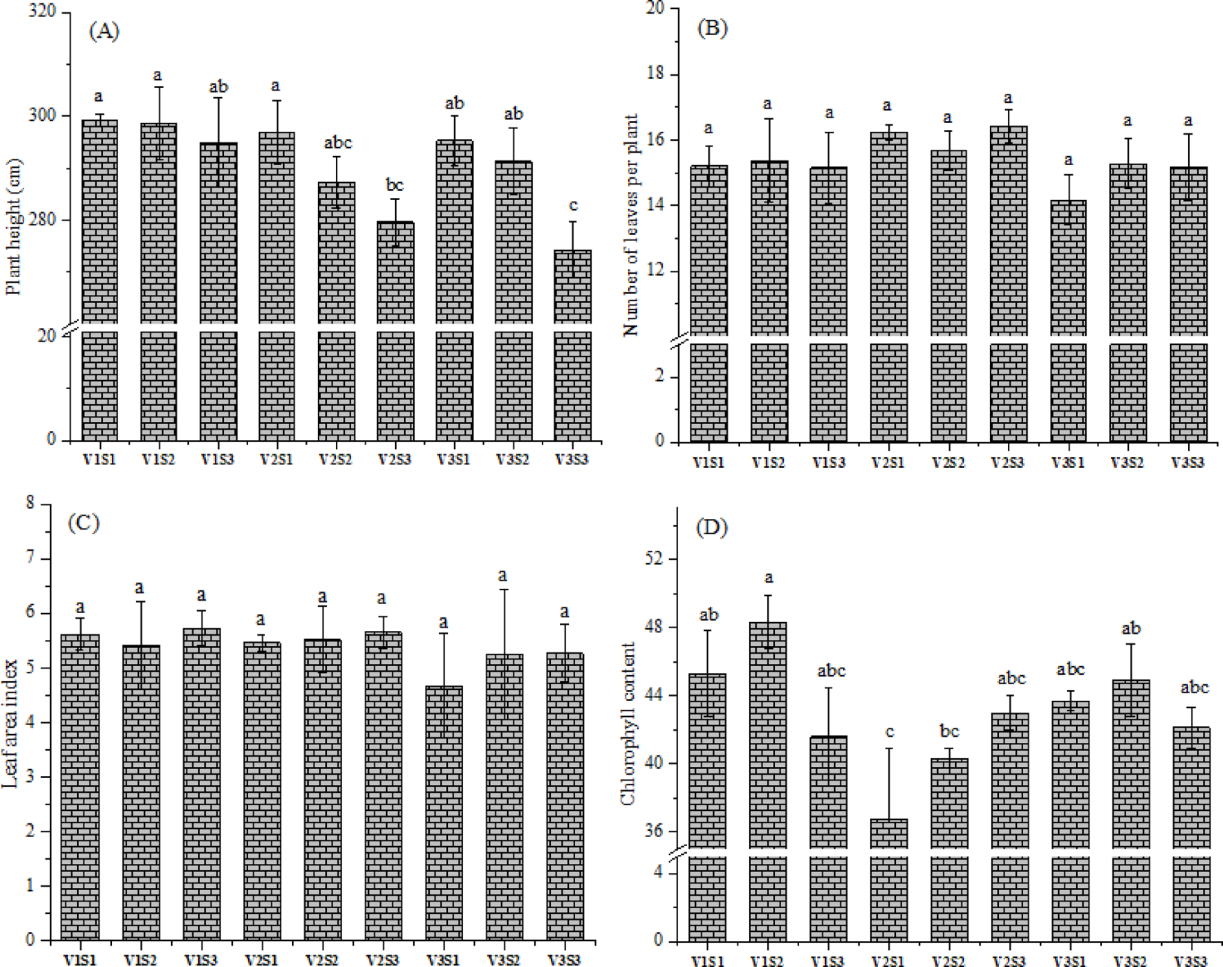
Interactive effects of hybrids and intra-row spacing levels on plant height (**A**), number of leaves per plant (**B**), leaf area index (**C**), and chlorophyll content (**D**) of corn. V1 = Pioneer 3444, V2 = Hytech 2031, V3 = Giza 168, S1 = 15 cm, S2 = 25 cm, S3 = 35 cm. Different letters on bars indicate significant differences according to Tukey’s test at p < 0.05.

Plant height is recognized as an imperative factor in determining the growth and yield of agricultural crops. In this regard, the effect of hybrid, intra-row spacing, and their interactions on plant height of three corn hybrids was examined. There were no remarkable differences in plant height of corn hybrids (p > 0.05) (Table 3), consistent with the investigation of Sibonginkosi et al.^32^. Pioneer 3444 (V1) exhibited tallest plants of 297.67 cm, followed by Hytech 2031 (V2) and Giza 168 (V3) with height of 288.00 and 287.00 cm, respectively. This may be attributable to variations in environmental or soil conditions. Moreover, plant height increased with reducing plant spacing (from 15–35 cm). Specifically, corn sown at 15 cm had significantly taller plants (297.22 cm) than those grown at 35 cm (283.00 cm; p < 0.05), while exhibiting no significant variation from those at the 25 cm spacing (292.44 cm; p > 0.05). Implicit from this is that, intra-row spacing dramatically affected plant height of corn hybrids throughout the evaluation period**.** The observed phenomenon was basically ascribed to intensified competition among plants for sunlight, which likely stimulated the vertical growth of plants to maximize light absorption^33^**.** Similarly, Miko and Manga^34^ documented an increase in the height of corn plants under narrower intra-row spacing. Yirzagla et al.^35^ also reported that narrower intra-row spacing resulted in taller plants, whereas wider spacing produced shorter plants. Contrarily, Ibeawuchi et al.^12^ noticed that plots sown at narrower spacing displayed the shortest plants, attributing this observation to heightened interplant competition for critical growth resources such as carbon dioxide and nutrients, along with limited access to other growth-promoting stimuli. Importantly, the study revealed a notable interaction between corn hybrids and plant spacing in terms of plant height. The V1 hybrid grown at a 15 cm spacing showed the tallest plants (299.33 cm), whereas the combination of V3 hybrid and 35 cm spacing resulted in the shortest one (274.33 cm) (Fig. 4A). These results were due to competition amongst plants for light, water, and nutrients under narrower spacing (15 cm). This may have prompted stem elongation (a shade avoidance response) to outcompete neighboring plants, resulting in greater height observed in the V1 hybrid. However, wider spacing (35 cm) reduces competition, allowing for better light distribution, but may also result in less stem elongation stimulus, leading to shorter plants in the V3. Similar investigations were also noticed by Muázu^36^.

**Table 3:** Effect of hybrids and intra-row spacing on growth attributes of corn. V1 = Pioneer 3444, V2 = Hytech 2031, V3 = Giza 168. S1 = 15 cm, S2 = 25 cm, S3 = 35 cm. Means in columns within groups followed by different letter(s) are statistically different according to Tukey’s test at p < 0.05. “ns” = non-significance, “*” = significance at p < 0.05, and “**” = significance at p < 0.01.

Treatment	Plant height (cm)	Number of leaves per plant	Leaf area index (LAI)	Chlorophyll content
A- hybrids (V)				
V1	297.67 ± 1.90^a^	15.23 ± 0.09^b^	5.59 ± 0.13^a^	45.09 ± 2.71^a^
V2	288.00 ± 7.09^a^	16.11 ± 0.32^a^	5.55 ± 0.08^a^	40.02 ± 1.57^b^
V3	287.00 ± 9.10^a^	14.87 ± 0.50^b^	5.06 ± 0.28^b^	43.58 ± 1.14^ab^
B- intra-row spacing (S)				
S1 (15 cm)	297.22 ± 1.64^a^	15.20 ± 0.84^a^	5.25 ± 0.42^a^	41.90 ± 3.72^a^
S2 (25 cm)	292.44 ± 3.70^ab^	15.44 ± 0.17^a^	5.40 ± 0.11^a^	44.52 ± 3.27^a^
S3 (35 cm)	283.00 ± 6.76^b^	15.58 ± 0.60^a^	5.55 ± 0.20^a^	42.27 ± 0.55^a^
Analysis of variance (F-test)				
V	3.3394 ns	5.87796*	2.6462 ns	8.3977**
S	5.0406*	0.5439 ns	0.4131 ns	2.5025 ns
V x S	0.7710 ns	1.0530 ns	0.5760 ns	3.4836*

### Number of leaves per plant

The number of leaves and their distribution ratio below to above the primary ear serve as key determinants of plant architecture. The single factor of hybrid notably affected the number of leaves per plant (Table 3). Number of leaves was observably higher in V2 hybrid than V1 and V3 (p < 0.05), while V1 and V3 were statistically similar (p > 0.05). This suggests that corn hybrids have different ability in using the growth space and adaptation process to environment^37^. Furthermore, the three spacing intervals showed no significant differences on number of leaves of corn. Although plants at 35 cm spacing had numerically more leaves (15.58) than those cultivated at 15 cm (15.20) and 25 cm (15.44), these differences were not statistically significant. Comparable observations were previously indicated by Belay et al.^38^. However, Ali et al.^39^ reported that densely spaced corn plants produced more leaves compared to those cultivated at wider spacing, and they linked this outcome to the accelerated growth rate in search for sunlight, space, nutrients, and other environmental resources. The synergistic effect of hybrids and plant spacing, however, had no notable impact on number of leaves per plant (p > 0.05) (Fig. 4B).

### Leaf area index (LAI)

LAI in corn plays a vital role in governing total light interception, which in turn influences main physiological processes such as photosynthesis, transpiration, and biomass accumulation. Table 3 revealed that V1 and V2 possessed significantly greater LAI than V3 (p < 0.05), but no observable difference existed among V1 and V2 (p > 0.05). The maximal LAI was recorded in V1 hybrid (5.59), followed by V2 (5.55) and V3 (5.06). The higher LAI observed in V1 hybrid reflects its superiority in leaf development, canopy efficiency, and sustained leaf area retention under competitive conditions. Additionally, this hybrid may be better suited for cultivation (over other hybrids) under high-input systems (e.g., optimal water and nutrient availability), where these traits synergistically maximize leaf production. Similar conclusions were previously indicated^40^. However, Sibonginkosi et al.^32^ realized no remarkable difference in LAI between corn cultivars. Moreover, LAI improved with increased plant spacing level. Nonetheless, plants grown at wide intra-row spacing (35 cm) had no significant increase in LAI compared to those cultivated at 15 and 25 cm (p > 0.05). These findings were in good line with those recorded by Ngugi et al.^41^, who indicated that lower plant population, by allowing greater access to nutrients and water, increased LAI compared to high plant density. Furthermore, the results illustrated that the interaction between hybrid and spacing had no significant (p > 0.05) effect on LAI (Fig. 4C).

### Chlorophyll content

Chlorophyll, the primary photosynthetic pigment, plays a fundamental role in photosynthesis by directly determining photosynthetic efficiency. This in turn influences crop growth and yield. As the dominant reservoir of leaf nitrogen, chlorophyll degradation during senescence impairs photosynthetic capacity, reducing light capture, limiting carbon fixation, and ultimately decreasing corn productivity^42,43^. Data showed that the highest chlorophyll content (45.09) was observed in V1 hybrid, which differed observably (p < 0.05) from V2 (40.02) (Table 3). This genotypic variation was mainly attributed to differences in photosynthetic efficiency, nutrient uptake capacity, or stress tolerance. The superior chlorophyll content in V1 was probably associated with improved pigment biosynthesis, optimized light-harvesting efficacy, and augmented nitrogen use capacity. Conversely, V3 hybrid depicted no considerable differences relative to V1 and V2 genotypes. The lower chlorophyll level observed in V2 may reflect impaired photosynthetic activity, insufficient nutrient assimilation, or heightened susceptibility to environmental stressors. This was in good conformity with the findings of Sadeghi^44^ and Zhang et al.^45^. Furthermore, intra-row spacing alone had no significant effect on chlorophyll content. Nonetheless, a numerical trend was observed where chlorophyll content increased and then decreased with increasing plant spacing (from 15 to 35 cm), reaching its peak value (44.52) at 25 cm. Notably, chlorophyll content varied significantly (p < 0.05) due to the interaction between hybrids and intra-row spacing level (Fig. 4D). The V1 hybrid cultivated at 25 cm showed the maximal chlorophyll content (48.30), while minimal content (36.73) was recorded for V2 under 15 cm spacing. This observation suggested that V1 at 25 cm spacing may have maximized solar radiation interception and utilization, thereby enhancing photosynthetic efficiency^46^.

### Days to 50% tasseling

**Fig. 5:**
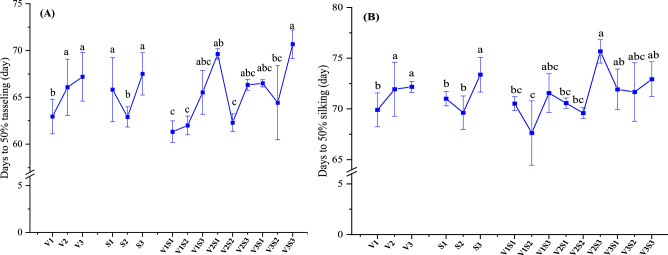
Effect of hybrids and/or intra-row spacing on days to 50% tasseling (A) and silking (B) of corn. V1 = Pioneer 3444, V2 = Hytech 2031, V3 = Giza 168, S1 = 15 cm, S2 = 25 cm, S3 = 35 cm. Different letters on bars indicate significant differences according to Tukey’s test at p < 0.05 within groups (V, S, and V × S).

Figure 5A shows the influence of hybrid, intra-row spacing, and their interaction on days to 50% tasseling in corn. V2 and V3 hybrids required significantly more days to reach 50% tasseling over V1 (p < 0.05). V3 exhibited the longest days to 50% tasseling (67.20 days), whereas the shortest (62.95 days) was documented for V1. The observed variations may be correlated with the differences in photoperiod sensitivity, and stress adaptation among hybrids^14^. Besides, the plant spacing depicted notable (p < 0.05) effect on days to 50% tasseling. The longest days to 50% tasseling (67.51 days) were noted at 35 cm spacing, followed by 15 cm (65.82 days) and 25 cm (62.91 days). This demonstrated that plants grown at 25 cm spacing achieved accelerated reproductive maturity, as evidenced by early tasseling, rather than prolonging the vegetative growth phase. In that sense, intra-row spacing (at 25 cm) may have intensified the photosynthesis rate (consistent with the outcome of chlorophyll content at the same spacing in this study), which accelerated the phenological development stages including tasseling. Wondimkun^10^ observed that the optimum plant spacing (35 cm) shortened the time to tasseling initiation relative to wider intra-row spacing (40 cm). However, Park et al.^47^ indicated that plant density had no impact on days to tasseling. On the other hand, the interaction between hybrids and plant spacings resulted in significant (p < 0.05) differences on days to tasseling. The V1 hybrid at 15 cm spacing displayed earliest tasseling (61.33 days). The longest duration to tasseling (70.67 days) was recorded for V3 hybrid at 35 cm followed by V2 hybrid at 15 cm (69.63 days). This outcome may have resulted from intensified resource competition under narrower spacing and greater resource availability in widely spaced plants.

### Days to 50% silking

The silking stage is a crucial period for grains formation following pollination. Figure 5B demonstrates that the V1 exhibited significantly different days to 50% silking relative to other hybrids (p < 0.05). No significant differences were noted between V3 and V2 (p > 0.05). This was in good accordance with the observations of days to 50% tasseling (Fig. 5A). The longest duration to 50% silking (72.18 days) was recorded for the V3 hybrid, whilst the V1 hybrid showed the shortest period (69.91 days). This may have resulted from the variations in maturity period among the hybrids. The observed conclusion was in good agreement with the investigations of Temesgen and Kebena^48^ and Ngugi. et al.^41^, who documented substantial differences in period to silking among corn genotypes. Furthermore, plant spacing displayed significant affected silking time. Plants at 15 cm (71.01 days) and 25 cm (69.63 days) silked significantly earlier than at 35 cm (73.38 days) (p < 0.05). Wondimkun^10^ also observed that densely spaced plots significantly shortened the time required for silk emergence of corn. However, Temesgen^14^ found that the changes in plant spacings had no significant effect on the days to 50% silking in corn (p > 0.05). This could be linked to the differences in corn hybrids and environmental conditions. Moreover, days to 50% silking also showed remarkable differences (p < 0.05) due to the synergistic effects between hybrids and spacing. The V2 hybrid at 35 cm spacing required the longest period (75.67 days) to reach 50% silking, whereas the shortest duration (67.63 days) was documented for V1 at 25 cm spacing. Such findings were also buttressed by the earlier outcomes of Dwipa et al.^24^, who indicated that the interaction between corn hybrids (Pioneer P35 and Pertiwi 3) and plant spacing levels (20–40 cm) significantly influenced the period to 50% silking.

### Ear length

**Fig. 6:**
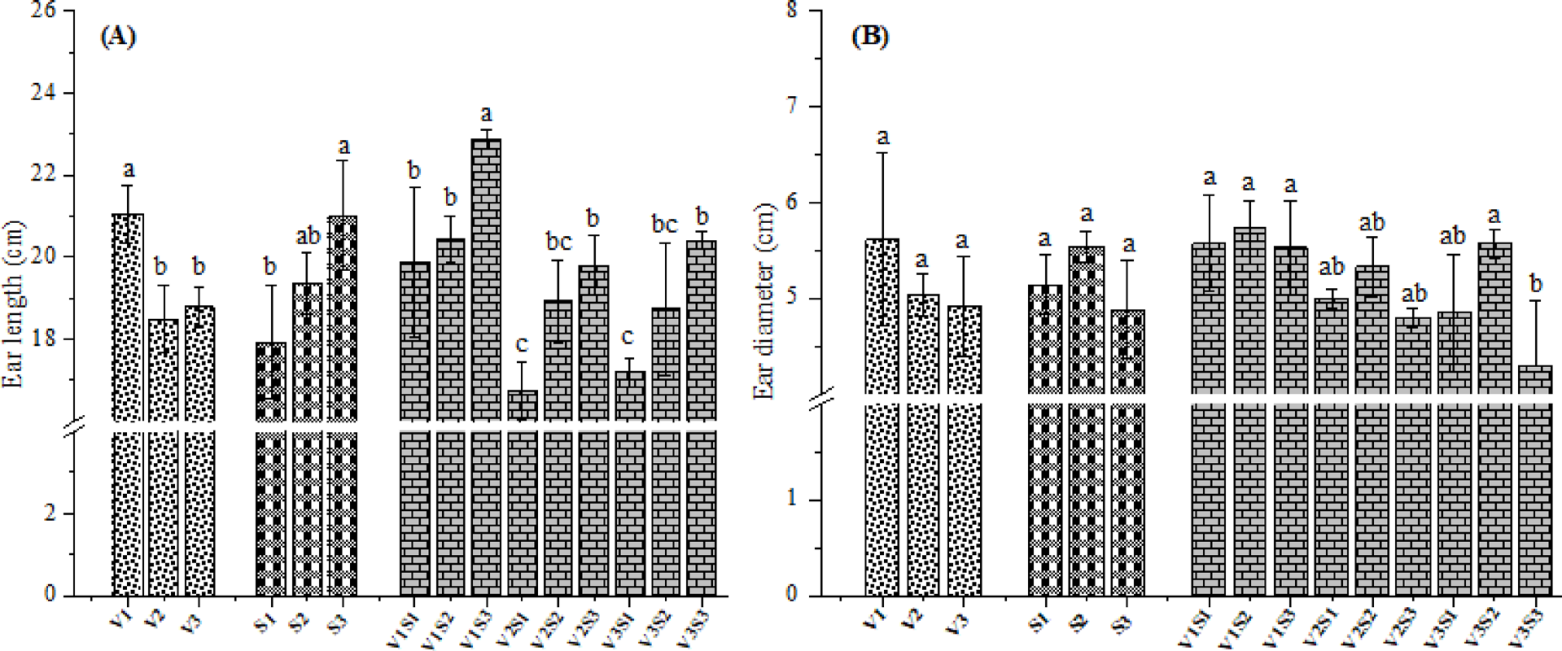
Effect of hybrids and/or intra-row spacing on ear length (**A**) and diameter (**B**) of corn. V1 = Pioneer 3444, V2 = Hytech 2031, V3 = Giza 168, S1 = 15 cm, S2 = 25 cm, S3 = 35 cm. Different letters on bars indicate significant differences according to Tukey’s test at p < 0.05 within groups (V, S, and V × S).

Figure 6A portrays the effect of hybrid and/or intra-row spacing on the ear length. V1 produced significantly longer ears (by 12.20 and 10.83%, respectively) than those observed for V2 and V3. The shortest ears were recorded for V2 (18.49 cm) which was statistically similar to V3 (18.78 cm). This indicates that the V1 hybrid exhibits higher efficiency in converting growth resources into economically valuable yield components and effectively allocating assimilates to sink tissues. Maintaining high assimilate availability throughout the corn grain-filling phase is crucial to maximize ear length^49^. The observed phenomenon was likely attributed to favorable environmental conditions that intensified the utilization of solar radiation in V1 (over other hybrids), strengthening the assimilate synthesis, and its effective conversion into starch, collectively increased ear length. Similarly, Abuzar et al.^50^ recorded significant variations in ear length among corn hybrids. Mandić et al.^8^ also found that plots sown with the AS534 hybrid exhibited ears that were 7.07% longer than those of NS5010 (p < 0.05), demonstrating significant variation in ear length among corn hybrids. Furthermore, intra-row spacing significantly affected ear length of corn plants (p < 0.05). However, no notable variation existed between 15 and 25 cm intra-raw spacings (p > 0.05). Corn plants sown at 35 cm spacing possessed the longest ears (21.02 cm), while the shortest ears (17.93 cm) were noticed in densely spaced plots (15 cm). Sabo et al.^51^ reported that plants grown at 25 cm spacing produced longer ears than those at 30 cm. Similarly, Zamir et al^52^ demonstrated a positive correlation between plant spacing and ear length of corn, linking this outcome to greater resources availability and reduced interplant competition at low plant population. Besides, the interaction of hybrids × intra-row spacing levels considerably (p < 0.05) affected the ear length. The V1 hybrid sown at spacing of 35 cm maximized the length of ears (22.87 cm), while the shortest ears (16.73 cm) were produced from V2 grown at 15 cm. Reduced ear length under narrow row spacing is mostly linked to limited assimilate availability caused by decreased photosynthetic activity in leaves, stemming from restricted access to growth-influencing factors^53^.

### Ear diameter

Figure 6B depicted that the effect of plant spacing or corn hybrids had no notable impact on ear diameter (p > 0.05). The V1 hybrid produced the largest ear diameter (5.61 cm), followed by V2 (5.04 cm) and V3 (4.91 cm) hybrids. Furthermore, plants grown at spacing of 25 cm had larger ear diameter by 7.22 than at 15 cm. However, the smallest ear diameter (4.88 cm) was found at 35 cm spacing. Hasan et al.^4^ also recorded the maximum ear diameter at 25 cm. Yirzagla et al.^35^ found that ear diameter increased and then decreased with increasing intra-row spacing (20–40 cm) (p > 0.05), reaching the maximal diameter at 30 cm spacing. Most importantly, the interaction amongst hybrids and spacing levels substantially affected ear diameter (p < 0.05). The largest diameter (5.73 cm) was produced where V1 was sown at 25 cm, while the smallest diameter (4.30 cm) was recorded form V3 × 35 cm spacing. Ear diameter is directly influenced by the allocation of photosynthetic assimilates to the ear. Greater assimilate allocation to ear correlated with larger ear diameter, a process driven by the redistribution of photosynthetic products from leaves and stems throughout grain filling. Increased assimilate availability in vegetative tissues (leaves and stems) enhanced the transfer of these resources to developing grains, thereby augmenting ear diameter^54^. The decrease in ear diameter of the V3 hybrid under wider intra-row (i.e., 35 cm) spacing was likely due to reproductive plasticity and changes in resource allocation, potentially resulting from reduced pollen-to-silk synchrony.

### Number of grains per row

**Fig. 7:**
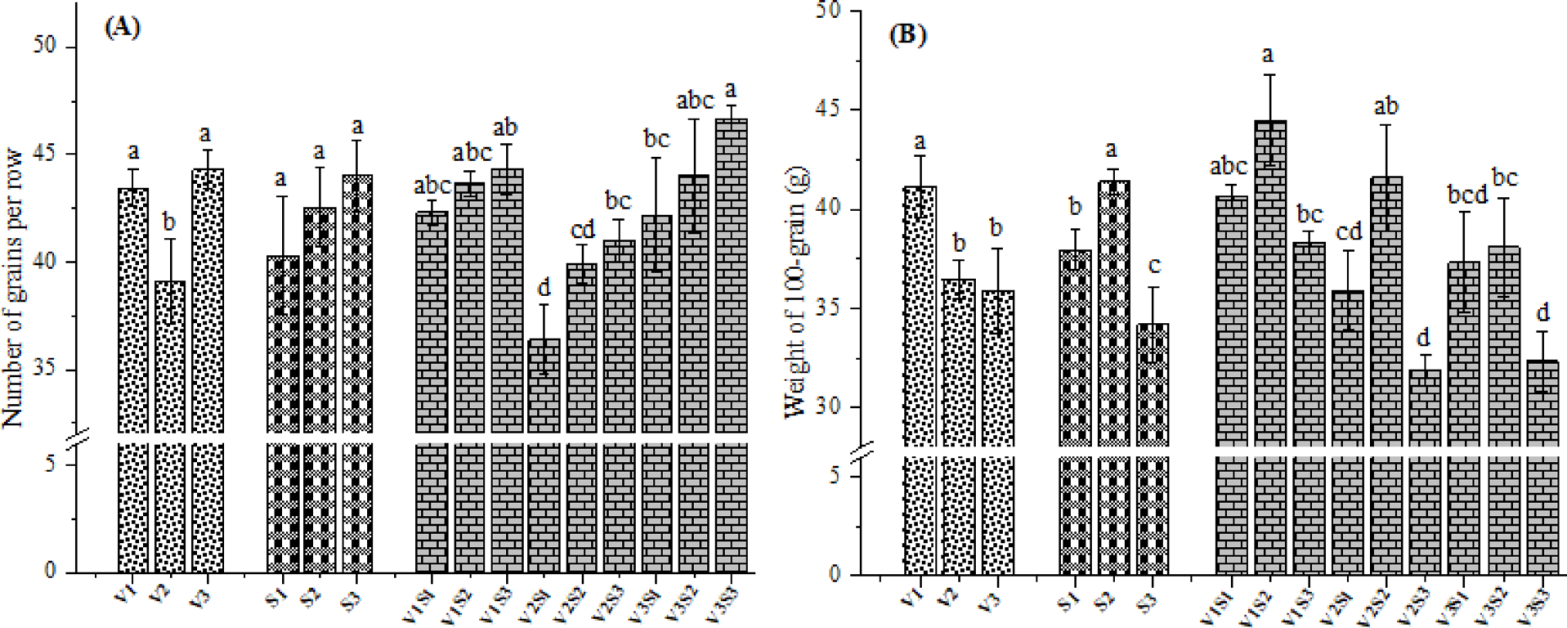
Effect of hybrids and/or intra-row spacing on number of grains per row (**A**) and weight of 100-grain (**B**) of corn. V1 = Pioneer 3444, V2 = Hytech 2031, V3 = Giza 168, S1 = 15 cm, S2 = 25 cm, S3 = 35 cm. Different letters on bars indicate significant differences according to Tukey’s test at p < 0.05 within groups (V, S, and V × S).

Number of grains per row directly contributes to the economic yield and reflects the productive efficiency in crop hybrids. Number of grains per row was significantly affected by the hybrid (p < 0.05). Specifically, V3 and V1 hybrids produced significantly more grains per row (44.29 and 43.44, respectively) compared to V2 (39.11) (Fig. 7A). Nevertheless, no significant difference was registered between V1 and V3 hybrids. Similar observations were recorded in literature^55^. With respect to plant spacing, the number of grains per row increased with increasing plant spacing, peaking at 35 cm (44.00). The observed outcome was probably attributable to optimal availability of growth-promoting resources, which enhanced plant growth and development. This likely improved light interception by the canopy, creating an early sink for assimilates, resulting in more grains per row. Kumar^56^ also reported that wider plant spacing impaired interplant competition and intensified photosynthetic efficiency, thereby enhancing the source-sink relationship, and subsequently increased ear size, number of rows per ear and number of grains per ear. Moreover, number of grains per row was significantly affected by the mutual impact of hybrids and plant spacing (Fig. 7A). The maximum number of grains per row was produced by V3 at 35 cm (46.67). The lowest value was attained from V2 × 15 cm spacing (36.40). Such variations highlight how optimal spacing varies among hybrids, shaped by their adaptation to planting density stress. Deducing from this is that, the 35 cm spacing likely aligned with the growth and physiological requirements of the V3, while the 15 cm exceeded the crowding tolerance threshold of the V2 hybrid.

### Weight of 100-grain

The impact of hybrids and/or intra-row spacing on weight of 100-grain is presented in (Fig. 7B). The heaviest weight of 100-grain (41.16 g) was observed in the V1 hybrid, followed by V2 (36.46 g) and V3 (35.91 g). The V1 exhibited a significant difference relative to other hybrids (p < 0.05), but there was no significant variation between V2 and V3 (p > 0.05). Comparable results were also reported by Mandić et al.^8^, who observed significant differences in weight of 1000-grain among corn hybrids. Moreover, notable changes in weight of 100-grain were recorded under varied intra-row spacing levels (p < 0.05). Plants grown at 25 cm spacing produced remarkably heavier weight by 8.29% reference to those at 15 cm. This illustrated that widely spaced plants reduced interplant competition, leading to better growth and thus contributed to the increase in weight of 100-grain over densely spaced plants. Additionally, the reduced 100-grain weight under narrower spacing was attributed to limited photosynthetic assimilate availability for grain development resulting from intense competition among plants. This may have impaired photosynthetic efficiency and augmented respiration rate due to enhanced mutual shading^52^. However, overly wide spacing (35 cm) yielded the lowest 100-grain weight (34.19 g), primarily due to inefficient resource use, excessive vegetative growth, poor pollination, and reduced light interception, all negatively impacting grain filling. A comparable trend was also reported elsewhere^57^. The interactive effects of hybrids and intra-row spacing was found to be significant on weight of 100-grain. The heaviest weight (44.50 g) was realized at 25 cm spacing and V1 hybrid, while the minimum weight (31.92 g) was produced by V2 at spacing of 35 cm. These results are in conformity with those observed by Gozubenli et al.^58^.

### Grain yield

**Fig. 8:**
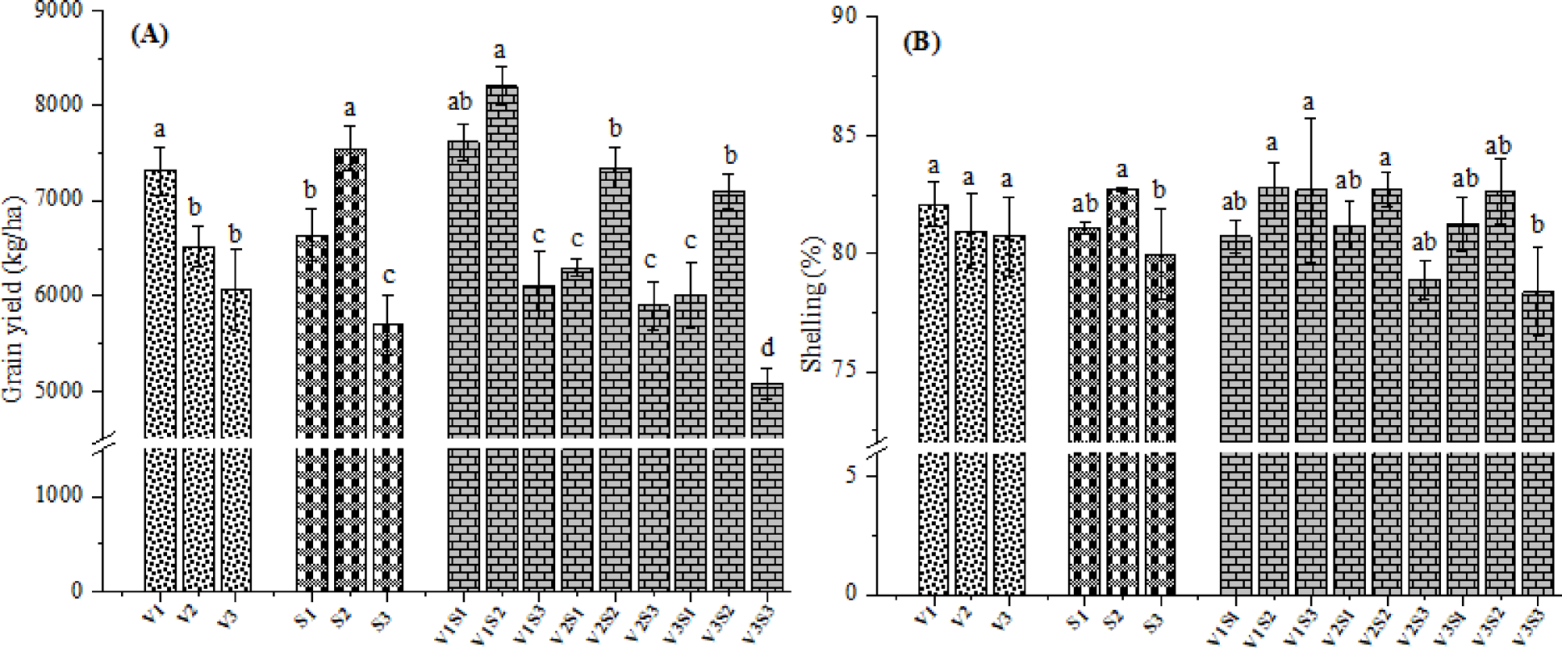
Effect of hybrids and/or intra-row spacing on grain yield (**A**) and shelling percentage (**B**) of corn. V1 = Pioneer 3444, V2 = Hytech 2031, V3 = Giza 168, S1 = 15 cm, S2 = 25 cm, S3 = 35 cm. Different letters on bars indicate significant differences according to Tukey’s test at p < 0.05 within groups (V, S, and V × S).

Grain yield depends on biomass accumulation and the proportion allocated to the grains, mainly linked to key agronomic factors such as plant density, number of ears per plant, number of grains per row, number of rows per ear, ear length and diameter, weight of 1000-grain, etc. In this regard, the variations in aforementioned factors have a direct impact on crop yield. The impact of hybrids, intra-row spacing, and their interactions was significant (p < 0.05) on grain yield (Fig. 8A). Data revealed that V1 hybrid showed significantly higher yield (7311.09 kg/ha) than V2 and V3 (6513.89 and 6061.54 kg/ha, respectively). This may be associated with the special qualities linked to the intrinsic traits of V1 hybrid, including disease resistance, early maturity, uniform flowering and ear positioning, and high yield potential^59^. Plants grown at spacing of 25 cm were remarkably superior (p < 0.05) in grain yield (7549.78 kg/ha), followed by plants grown at 15 and 35 cm (6640.43 and 5696.40 kg/ha, respectively). Such findings were consistent with ear diameter and weight of 100-grain in this research. Bisht et al.^60^ also found that plant population exceeding optimal levels caused lodging, which subsequently reduced corn production. The observed results may also be explained by a higher number of harvestable ears achieved through optimal spacing. This was supported by the findings of Sener et al.^61^ and Eskandarnejada et al.^62^, who observed that grain yield per plant augmented with wider inter- and intra-row spacing and decreased under narrower spacing. A notable reduction by 12.04% in grain yield was recorded under low intra-row spacing (15 cm) compared to plants grown at 25 cm. This reduction was primarily due to the fact that high plant density (i.e., low intra-row spacing) exceeding the optimal limit increased plant infertility and unevenness, mainly by extending the pollen-shedding-to-silking interval^16^. This resulted in more unproductive plants and lower final yield, consistent with the outcomes of duration to 50% silking (Fig. 5B). Surprisingly, the minimal yield was documented when corn was sown at 35 cm. Excessively wide plant spacing promotes weed proliferation and impairs light interception and nutrient uptake, as excess sunlight reaches the soil surface and the nutrients are lost via evaporation and leaching. Additionally, wider spacing reduces the number of plants per ha, potentially lowering total grain yield. Besides, wider spacing not only causes inefficient resource use (e.g., fertilizers and irrigation) but also reduces potential productivity by limiting optimal plant density. Andrade et al.^63^ found a linear association among corn growth and intercepted photosynthetically active radiation (PAR). As a result, plant population influences PAR and radiation use efficacy (RUE) during the grain-filling period, which in turn affects kernel weight and final grain yield. Considering the interaction effect of both hybrid and plant spacing, corn hybrids greatly varied in their response to decreased or increased intra-row spacings, with changes in grain yield. The highest grain yield (8206.87 kg/ha) was produced from V1 hybrid grown at 25 cm spacing and the lowest yield (5078.72 kg/ha) was noted from V3 cultivated at 35 cm. This finding was also supported by Sabo et al.^51^, who indicated that plants grown at spacing of 25 cm exhibited better performance than those cultivated at 20 and 30 cm. The increase in yield was attributable to enhanced dry matter production during grain filling, buttressed by increased photosynthetic capacity due to a greater number of leaves^64^.

### Shelling percentage

Corn shelling is the process of removing grains from the ear. The shelling percentage, a crucial parameter in corn production, reflects the ratio of edible grains removed from the ear. It is strongly influenced by several factors, including the determination method, agro-climatic conditions, genotypes, cultural practices, and moisture content of grain^65^. Results indicated that highest shelling percentage (82.08%) was documented for the V1 hybrid, whilst the lowest was recorded for the V3 (80.75%) (Fig. 8B). Moreover, intra-row spacing led to significant variations in shelling percentage. The maximal shelling percentage (82.72%) was observed at 25 cm, showing a significant difference compared to wider spacing (35 cm; 79.98%). However, plants spaced at 15 cm (81.06%) were statistically at par with those at 25 and 35 cm (p > 0.05). The observed results are in agreement with those of Ogunlela et al.^66^ and Mukhtar et al.^67^. Observably, Fig. 8B demonstrates a significant interaction between corn hybrids and plant spacing on shelling percentage. The V1 hybrid cultivated at 25 intra-rows spacing achieved the maximal shelling percentage (82.83%), while the mutual effect of the V3 hybrid and 35 cm spacing produced the lowest value (78.39%).

### Total carbohydrate content

**Fig. 9:**
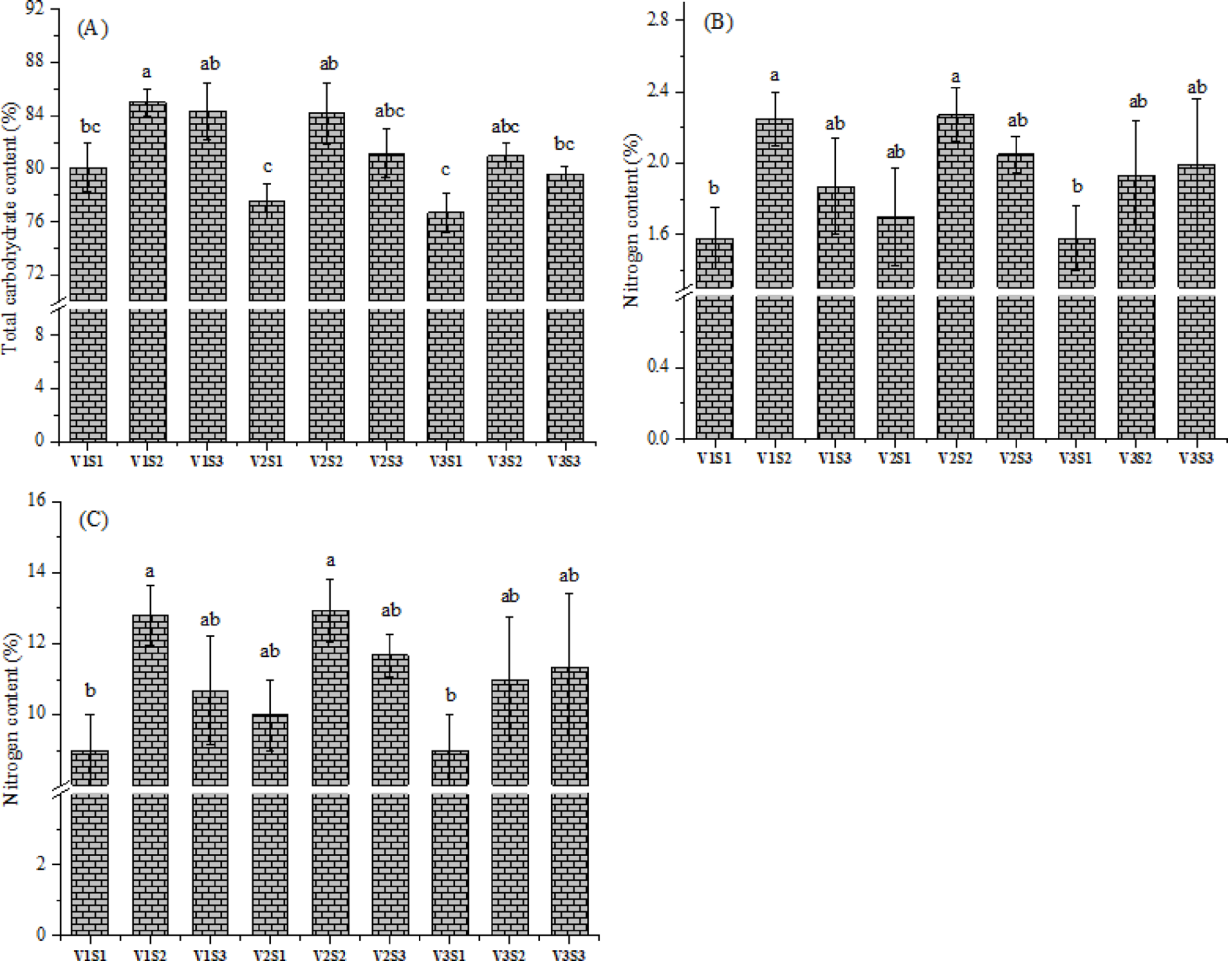
Interactive effects of hybrids and intra-row spacing levels on total carbohydrate content (**A**), Nitrogen content (**B**), and protein content (**C**) of corn. V1 = Pioneer 3444, V2 = Hytech 2031, V3 = Giza 168, S1 = 15 cm, S2 = 25 cm, S3 = 35 cm. Different letters on bars indicate significant differences according to Tukey’s test at p < 0.05.

Corn is widely recognized as a carbohydrate-rich crop, making it a valuable energy source. Variation in carbohydrate composition among the hybrids highlights the potential for targeted breeding programs to enhance nutritional profiles^68^. Results (Table 4) showed significant differences in carbohydrate content among the three selected corn hybrids (p < 0.05). Carbohydrates content ranged from 79.10 to 83.14%, and the maximum content was realized for the V1 hybrid. The V3 hybrid produced the lowest carbohydrate content. Such results were also comparable to what was previously reported^69^. Moreover, the outcomes depicted that the changes in plant spacing levels notably affected carbohydrate content (p < 0.05). Specifically, plants spaced at 25 cm portrayed better accumulation of carbohydrates (83.40%) than those cultivated at 15 and 35 cm (p < 0.05). However, no considerable difference in carbohydrate content was noticed amongst plants grown at 15 and 35 cm (p > 0.05). Deducing from this is that, optimum spacing (i.e., at 25 cm) may have promoted adequate light penetration, efficient nutrient distribution, and improved root development. This may have boosted the photosynthetic rate and maximized assimilate allocation to developing kernels, resulting in increased starch and soluble sugar content while minimizing competition among plants. Duvick^70^ also revealed that dense planting (i.e., higher plant density) enhanced the capacity of corn plants to optimize solar radiation interception earlier in the growth stages, transforming this energy into storage carbohydrates. Moreover, statistical analysis indicated that carbohydrate content was substantially affected by the interaction between hybrids and intra-row spacing (Fig. 9A). V1 sown at 25 cm spacing exhibited the highest carbohydrate content 85.00%, whereas V3 hybrid grown at 15 cm recorded the least content (76.67%). This suggests that the 25 cm spacing enabled V1 plants to intercept solar radiation more efficiently (compared to other combined effects), ultimately converting this energy into storage carbohydrates, thereby maximizing carbohydrate content.

**Table 4:** Effect of hybrids and intra-row spacing on chemical analysis of corn. V1 = Pioneer 3444, V2 = Hytech 2031, V3 = Giza 168, S1 = 15 cm, S2 = 25 cm, S3 = 35 cm. Means in columns within groups followed by different letter(s) are statistically different according to Tukey’s test at p < 0.05. “ns” = non-significance, “*” = significance at p < 0.05, and “**” = significance at p < 0.01.

Treatment	Total carbohydrate content (%)	Nitrogen content (%)	Protein content (%)
A- hybrids			
V1	83.14 ± 2.17^a^	1.90 ± 0.27^a^	10.82 ± 1.56^a^
V2	80.98 ± 2.71^b^	2.01 ± 0.23^a^	11.53 ± 1.20^a^
V3	79.10 ± 1.81^c^	1.83 ± 0.18^a^	10.44 ± 1.03^a^
B- intra-row spacing			
S1 (15 cm)	79.52 ± 2.24^b^	1.62 ± 0.06^b^	9.33 ± 0.47^b^
S2 (25 cm)	83.40 ± 1.13^a^	2.15 ± 0.16^a^	12.24 ± 0.88^a^
S3 (35 cm)	80.30 ± 0.64^ab^	1.97 ± 0.07^a^	11.22 ± 0.42^a^
Analysis of variance (F-test)			
V	20.5329**	2.5652 ns	3.4069 ns
S	23.6375**	25.4727**	24.3109**
V x S	8.6644**	1.4681 ns	1.5631 ns

### Nitrogen content

Nitrogen (N) is an essential nutrient for corn growth, directly impacting yield and biomass production. Intra-row spacing serves as a key determinant of nitrogen uptake, utilization efficiency, and overall crop productivity. The results also showed that the N content varied from 1.83 to 2.01% among the hybrids (p > 0.05) (Table 4). The V1 hybrid had the highest N content, whilst the minimal content was documented for V3. Furthermore, nitrogen content increased gradually from 1.97 to 2.15% as intra-row spacing widened from 15 to 25 cm (p < 0.05). It then decreased observably to 1.62% at the 35 cm spacing. Optimum plant spacing (25 cm) may reduce interplant competition for nitrogen, allowing better root expansion and development, augmented nutrient absorption per plant, improved light penetration and air circulation, indirectly enhancing photosynthetic efficacy and nitrogen utilization. Additionally, findings indicated that there was no significant effect on N content between the two plant distances of 25 and 35 cm (p > 0.05). The interaction between hybrids and intra-row spacing had stimulatory action on nitrogen content (Fig. 9B). The maximum nitrogen content (2.27%) was noticed in V2 hybrid cultivated at 25 cm, closely followed by the V1 at the same spacing (2.25%). However, V3 and V1 hybrids grown at the same spacing of 15 cm exhibited the lowest content (1.58 and 1.58%, respectively).

### Protein content

Protein plays an imperative role in nutritional value, plant growth, and industrial applications. The data indicated that protein content varied among the three corn hybrids (Table 4), but such differences were not statistically significant (p > 0.05). The V2 hybrid exhibited the highest protein content (11.53%), followed by V1 and V3 (10.82 and 10.44%, respectively). On the other hand, protein content was notably affected by plant spacing. Protein content increased substantially (p < 0.05) in plants grown at 25 cm and 35 cm intra-row spacing relative to closer spacing (15 cm). Nonetheless, no significant difference in protein content was noted among the 25 and 35 cm spacings (p > 0.05). These results were in agreement with those obtained by Zhang et al.^71^. The interaction between corn hybrids and intra-row spacing significantly influenced protein content (Fig. 9C). The analysis revealed that V1 and V2 hybrids cultivated at 25 cm intra-row spacing exhibited significantly higher protein content (12.93, and 12.80%) compared with V1 and V3 hybrids (9.00%, respectively) sown under the closer spacing of 15 cm. This suggests that V1 and V2 hybrids synthesized higher amino acid levels under optimal intra-row spacing (i.e., 25 cm), resulting in grains with enhanced nutritional quality.

### Correlation analysis

**Fig. 10:**
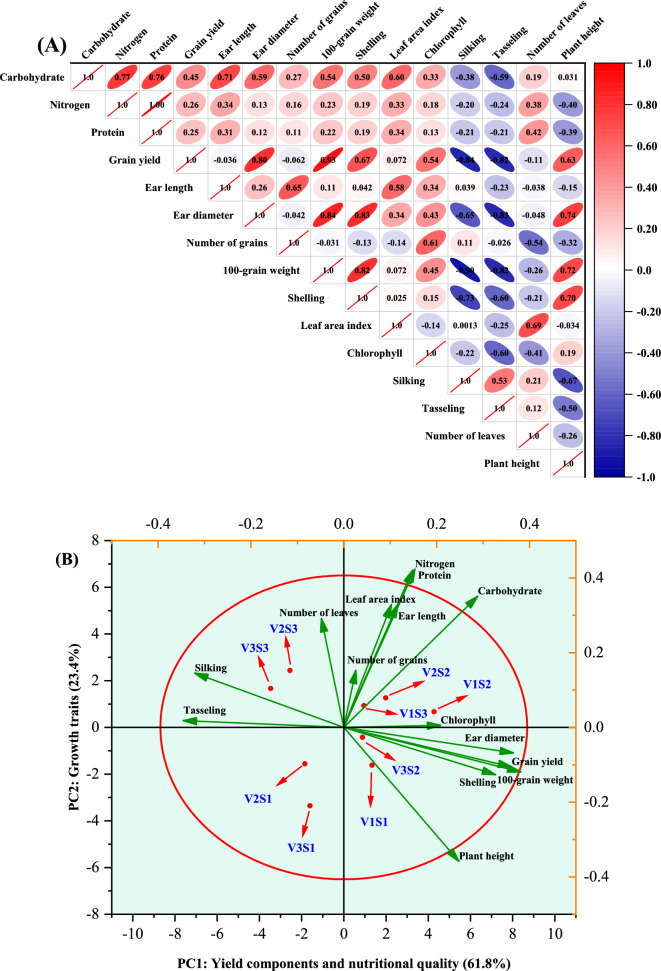
Correlational analysis (**A**) and PCA (**B**) of growth, yield, and chemical traits of corn hybrids sown under varied intra-row spacings. V1 = Pioneer 3444, V2 = Hytech 2031, V3 = Giza 168, S1 = 15 cm, S2 = 25 cm, S3 = 35 cm, V1S1 = Pioneer 3444 × 15 cm, V1S2 = Pioneer 3444 × 25 cm, V1S3 = Pioneer 3444 × 35 cm, V2S1 = Hytech 2031 × 15 cm, V2S2 = Hytech 2031 × 25 cm, V2S3 = Hytech 2031 × 35 cm, V3S1 = Giza 168 × 15 cm, V3S2 = Giza 168 × 25 cm, V3S3 = Giza 168 × 35 cm.

Pearson’s correlation (Fig. 10A) was employed to investigate how the growth, yield, and chemical characteristics of corn interrelate under the influence of hybrids and intra-row spacing. Results on plant height illustrated direct correlation with shelling percentage (*r* = 0.70), weight of 100-grain (*r* = 0.72), ear diameter (*r* = 0.74), and grain yield (*r* = 0.63), whilst exhibiting inverse relationship with days to 50% silking (*r* = -0.67). Such observations were possibly associated with a reduction in interplant competition under optimal intra-row spacing, which facilitated water and nutrient uptake for corn plants, stimulating a marked augmentation in grain yield. The observed increase in plant height is often correlated with alterations in canopy architecture, primarily through a more favorable leaf arrangement. This structural improvement enhances light interception efficiency by reducing mutual shading of leaves and increasing the capture of photosynthetically active radiation (PAR), particularly in the middle and lower canopy layers. This likely contributed to greater whole-canopy photosynthetic capacity during critical growth stages (e.g., silking and grain filling). The increased availability of photoassimilates (mainly sugars) would have directly supported stronger reproductive development in two key ways: 1) Pre-anthesis: an enhanced carbohydrate supply promotes the initiation and development of a greater number of florets, potentially leading to larger ears with more grain rows and more grains per row (i.e., increased grain sites). 2) Post-anthesis: a sustained photosynthetic capacity ensures a prolonged and ample supply of assimilates for grain filling. This directly enhances 100-grain weight (via complete and sustained filling), shelling percentage (through improved grain set efficiency), and ear size, ultimately increasing grain yield^38,72,73^. Besides, a positive correlation amongst number of leaves per plant and leaf area index was noticed (*r* = 0.69). The observed correlation between number of leaves per plant and leaf area index (LAI) stems from increased photosynthetic area per ground unit. More leaves directly increase LAI, enhancing light interception and photosynthetic efficiency^39^. Moreover, the heterotic canopy of the hybrids, characterized by more upright leaf angles, facilitates deeper light penetration and decreases self-shading, thereby reducing lower-leaf senescence and maintaining overall canopy photosynthesis. This subsequently boosts carbon assimilation, providing the energy and resources necessary to improve reproductive growth. Related associations between growth and yield properties of corn have previously been documented^9,59^. The prolonged period to 50% tasseling accounted strongly for 82–83% of the decrease in 100-grain weight, ear diameter, and grain yield, but with a corresponding increase (53%) in duration to 50% silking. Additionally, the increase in duration to 50% silking contributed negatively to shelling percentage (*r* = 0.73), weight of 100-grain (*r* = 0.90), and corn yield (*r* = 0.84); highlighting its detrimental influence on corn productivity. These significant negative correlations indicate that an extended duration to 50% silking prolongs the vegetative growth phase, delaying the shift in resource allocation from vegetative source tissues (leaves, stems) to reproductive sink development (the ear and grains). This delay can create a source-sink imbalance during the critical grain-filling period. Later-tasseling/ silking hybrids may face suboptimal environmental conditions (e.g., higher temperatures) that compromise photosynthetic efficiency and reduce the available assimilates. Conversely, early-tasseling/ silking hybrids synchronize their peak demand for photoassimilates (the sink strength of the grain) with a period of high photosynthetic capacity and more favorable environmental conditions. This optimal timing enhances the efficiency of photosynthate partitioning, directing a greater proportion of carbohydrates to the developing ear. This mechanism directly explains the observed improvements in shelling percentage (reduced grain abortion), 100-grain weight (complete grain filling), and ultimately, grain yield. Implicit from this is that, the changes in growth parameters, influenced by hybrid and spacing, significantly affected the grain productivity and yield-related attributes of corn. Importantly, direct correlations were realized between shelling percentage, ear diameter, weight of 100-grain, and grain yield (*r* = 0.67–0.93), consistent with the investigations of Wondimkun^10^ and Koirala et al.^21^. This finding implies that the augmentation in these yield components contributed positively to grain productivity. On the other hand, nitrogen and protein content were interrelated directly with total carbohydrate content (*r* = 0.76 and 0.75, respectively), illustrating good evidence for the intrinsic relationship among quality characteristics of corn grains. To end with, Pearson’s correlation demonstrated that growth characteristics of corn were associated strongly with both yield and its components.

To simplify the interpretation of interactive effects of hybrids and plant spacing on the growth, yield, and chemical traits of corn, PCA was performed (Fig. 10B). The PC1 and PC2 accounted for 85.2% of the overall variance (61.8% and 23.4%, respectively), capturing a sufficient amount of variance to reliably interpret the similarities and variations among the treatments^74^. The parameters and treatments clustered into four distinct groups. V1S1 and V2S3 were located in the positive and negative quadrants of both PC1 and PC2, respectively. These treatments were closely associated with the plant height, shelling percentage, 100-grain weight, grain yield, and ear diameter, suggesting that these hybrids and spacings markedly affected grain productivity and yield-related attributes. The second cluster (containing V1S3, V1S2, and V2S2), positioned in the upper right-hand quadrant, included chlorophyll, number of grains per row, ear length, leaf area index, carbohydrate content, nitrogen content, and protein content, reflecting strong associations with the V1 and V2 hybrids sown at wider spacing (25 and 35 cm). The third group contained V2S3 and V3S3 is situated respectively in the negative and positive side of PC1 and PC2, respectively. This group was interrelated with the number of leaves per plant and duration to 50% tasseling and silking. The observed outcomes imply that V2 and V3 cultivated at the same intra-row spacing (35 cm) contributed substantially to the improvement in growth traits of corn. Conversely, V2S1 and V3S1 lacked interrelationships with any of the measured parameters. Implicit from this is that, the hybrid and plant spacing had distinct and varied effects on the growth, yield, and quality attributes of corn.

## Conclusions

This research investigated the effect of hybrids and intra-row spacings on the growth, yield, and quality characteristics of corn. The findings demonstrated that the V1 displayed the tallest plants, as well as the highest LAI and chlorophyll content, whilst V3 required a longer duration to reach 50% tasseling and silking. Moreover, plant spacing at 25 cm increased yield and quality characteristics of corn relative to 15 and 35 cm spacings. Additionally, the maximal ear diameter, weight of 100-grain, grain yield, and carbohydrate content were observed for V1 hybrid at 25 cm. The V2 cultivated at 25 cm spacing displayed the highest nitrogen and protein content. The current outcomes indicate that farmers should cultivate the V1 hybrid at 25 cm spacing to achieve an optimal balance between corn yield and quality. In conclusion, while this study provides valuable insights into how hybrids and intra-row spacings affect corn growth, yield, and quality, its findings are context-specific to local soil properties (e.g., texture, fertility, pH) and climatic conditions (e.g., temperature, relative humidity). These factors significantly influence nutrient availability, water stress, and crop development rates, all of which can alter hybrid performance and optimal spacing recommendations. Therefore, future work should include multi-location trials across diverse agro-ecological regions to enhance the generalizability and translational potential of these findings. Furthermore, the absence of comprehensive economic analysis underscores the need for future research to validate practical recommendations for agricultural stakeholders.

## Data Availability

All data are included in this article, and any further information will be made available from the corresponding author on reasonable request.
